# MHC Class I Endosomal and Lysosomal Trafficking Coincides with Exogenous Antigen Loading in Dendritic Cells

**DOI:** 10.1371/journal.pone.0003247

**Published:** 2008-09-19

**Authors:** Genc Basha, Gregory Lizée, Anna T. Reinicke, Robyn P. Seipp, Kyla D. Omilusik, Wilfred A. Jefferies

**Affiliations:** 1 Biomedical Research Centre, Michael Smith Laboratories, Department of Microbiology and Immunology, University of British Columbia, Vancouver, British Columbia, Canada; 2 Biomedical Research Centre, Michael Smith Laboratories, Department of Zoology, University of British Columbia, Vancouver, British Columbia, Canada; 3 Biomedical Research Centre, Michael Smith Laboratories, Department of Medical Genetics, University of British Columbia, Vancouver, British Columbia, Canada; Institut Pasteur, France

## Abstract

**Background:**

Cross-presentation by dendritic cells (DCs) is a crucial prerequisite for effective priming of cytotoxic T-cell responses against bacterial, viral and tumor antigens; however, this antigen presentation pathway remains poorly defined.

**Methodology/Principal Findings:**

In order to develop a comprehensive understanding of this process, we tested the hypothesis that the internalization of MHC class I molecules (MHC-I) from the cell surface is directly involved in cross-presentation pathway and the loading of antigenic peptides. Here we provide the first examination of the internalization of MHC-I in DCs and we demonstrate that the cytoplasmic domain of MHC-I appears to act as an addressin domain to route MHC-I to both endosomal and lysosomal compartments of DCs, where it is demonstrated that loading of peptides derived from exogenously-derived proteins occurs. Furthermore, by chasing MHC-I from the cell surface of normal and transgenic DCs expressing mutant forms of MHC-I, we observe that a tyrosine-based endocytic trafficking motif is required for the constitutive internalization of MHC-I molecules from the cell surface into early endosomes and subsequently deep into lysosomal peptide-loading compartments. Finally, our data support the concept that multiple pathways of peptide loading of cross-presented antigens may exist depending on the chemical nature and size of the antigen requiring processing.

**Conclusions/Significance:**

We conclude that DCs have ‘hijacked’ and adapted a common vacuolar/endocytic intracellular trafficking pathway to facilitate MHC I access to the endosomal and lysosomal compartments where antigen processing and loading and antigen cross-presentation takes place.

## Introduction

In order to generate appropriate CD8^+^ T cell-mediated immune responses to viral, bacterial, self, or tumor-associated protein antigens, professional antigen-presenting cells (pAPCs) must acquire these antigens from the extracellular milieu, process them into antigenic peptides, load them onto MHC class I (MHC-I) molecules, and present them at the cell surface [1]–[4]. This process of “cross-presentation” is known to occur most efficiently in dendritic cells (DCs), but several mechanistic details remain unclear.

Three non-mutually exclusive pathways have been proposed to explain cross-presentation. In the vacuolar pathway, DCs internalize exogenous antigens and transport them through the endocytic pathway, where the internalized proteins are processed by cathepsins and other proteases into antigenic peptides [5], [6]. In this model, loading of peptides onto MHC-I molecules occurs directly within early and late endosomal/lysosomal compartments, often in a transporter associated with antigen processing-independent manner [7], [8]. In the endosome-to-cytosol pathway, internalized protein antigens are transported out of endosomes and into the cytosol by a mechanism that may involve ER-associated retrotranslocation complexes [9]–[12]. Subsequently, protein antigens follow the classical pathway of direct MHC-I presentation involving proteasome-mediated protein degradation and TAP transport of antigenic peptides into the endoplasmic reticulum (ER) for loading onto nascent MHC-I molecules [13]. A third proposed model of cross-presentation describes a unique intracellular compartment of exogenous antigen loading, termed the ergosome, which involves a possible fusion of ER with phagosomes containing internalized antigenic cargo [14], [15]. Although the biogenesis of ergosomes is a matter of some dispute [16], the concept of an ER-phagosome ‘mix’ compartment remains an attractive model for explaining where peptide-receptive MHC-I molecules could intersect with a relatively high concentration of exogenous antigens, presumably a crucial prerequisite for efficient cross-presentation.

MHC-I molecules have been reported to reside within endosomes and lysosomes of DCs [17], [18], but their source and intracellular trafficking routes have not yet been clearly defined. Although recycling between the cell membrane and endosomal compartments has been demonstrated for MHC-I in both in T cells and macrophages 19, 20, little is known about MHC-I endocytic trafficking in DCs or how this relates to their function in cross-presenation. We recently demonstrated a crucial role for a conserved, exon 6-encoded MHC-I cytoplasmic tyrosine in DC cross-presentation of exogenous antigens, and in the generation of *in vivo* cytolytic T lymphocyte responses against viruses [21]. Although we showed that mutation of the cytoplasmic tyrosine drastically reduced localization of MHC-I within endolysosomal compartments of DCs, the dynamic contribution of surface MHC-I to these compartments was not addressed. In this study, we define the mechanism whereby MHC-I molecules gain direct access to the intracellular compartment in DCs where peptide loading takes place. This mechanism underlies the unique ability of DCs to cross-present antigens and promote and initiate primary adaptive immune responses.

## Materials and Methods

### MHC class I internalization in dendritic cells

All experiments involving mice have been approved and performed in accordance with Canadian Council on Animal Care requirements. Splenic DCs were isolated as described (21),cultured overnight at 37°C in complete RPMI and the next day washed in PBS 3 times, aliquoted and labeled with Fc blocker 2.4G2 Fc_γ_ III/II (BD PharMingen) for 30 min at 4°C to exclude binding of antibodies to Fc receptor, followed by labeling with AF6-88.5 (H-2K^b^) specific monoclonal antibody conjugated to fluorescein isothiocyanate (FITC) for 30 min at 0°C in 96-well plates. Next, they were incubated at 37°C and at different time points as indicated, they were chilled to 4°C on ice before fixing in 2% paraformaldehyde. Recovered DCs were pipetted onto coverslips, mounted on slides and examined with a Nikon multiphoton immunofluorescent confocal microscope (ICM).

In order to assess the kinetics of internalization of MHC-I molecules, DCs were isolated as described, cultured at 37°C and the next day they were washed and stained with Fc blocker followed by AF6-88.5 (H-2K^b^) and 36-7-5 (H-2K^k^) antibodies (BD Biosciences, Mississauga, ON, Canada) conjugated to FITC and phycoerythrin (PE) respectively, at 4°C for 30 min in 96-well plates. Next, sample cells were placed at 37°C, whereas control cells were placed at 0°C and after different time points DCs were washed 3 times, fixed in ethanol to preserve FITC fluorescence and resuspended in 2% FCS PBS to reach an equal pH of 7.4 and prevent further quenching. DCs with fluorescently labeled H-2K^b^ molecules were examined using FACSCalibur™ (Becton Dickinson). Data were analyzed using FlowJo software to examine the H-2K^b^ and H-2K^k^ molecules following incubation at 37°C as an indication of MHC-I internalization.

### Intracellular DC colocalization and image quantification

Spleen-derived DCs from transgenic mice were aliquoted in 96-well plates and stained with H-2K^b^-FITC antibody after blocking the Fc receptor. Next, DCs were resuspended in 200 µL of 37°C pre-warmed completed RPMI, mounted onto Poly-D-lysine pre-coated coverslips and incubated at 37°C, allowing antibody-bound H-2K^b^ molecules to internalize. At different time points, as indicated, internalization was stopped by soaking the coverslips in cold PBS. At the end of the last time point, all coverslips containing DCs were treated with 2% BSA in PBS followed by fixation with 2% paraformaldehyde. Spleen-derived DCs were then permeabilized with 0.1% saponin in 2% BSA PBS followed by incubation with goat anti-mouse EEA1 or LAMP-1 primary antibodies (Santa Cruz Biotechnology, Santa Cruz, CA, USA). Secondary Alexa-568-conjugated rabbit anti-goat antibody (Molecular Probes, OR, USA) was used as a detection reagent. Isotype control antibodies were used in all confocal microscopy experiments to confirm the specificity of antibody staining. Images were acquired using a Nikon-C1, TE2000-U ICM and the EZ-C1 software. Data were analyzed using ImageJ.1 to select single slices and Adobe Photoshop 9.0 to merge images obtained from Red and Green channels.

To visualize the acquisition of exogenous OVA peptide by internalized H-2K^b^ molecules, DCs surface-labeled with H-2K^b^-FITC antibody as described above were incubated at 37°C in complete RPMI containing either 5 mg/mL ovalbumin protein (Worthington, NJ) or bovine serum albumin control protein. Next, DCs were mounted onto coverslips and incubated at 37°C for 6 hours, allowing ovalbumin protein uptake, processing and loading onto H-2K^b^ molecules. Incubation was halted by chilling the coverslips in ice-cold PBS. DCs were then fixed and permeabilized as described and following Fc blocking for 30 min, they were co-stained with goat anti-mouse EEA1, LAMP-1 or rat anti-mouse Giantin (Golgi marker) and with anti-H-2K^b^/OVA_257–264_ antibody. Goat anti mouse or Rabbit anti-goat coupled to Alexa-568 (Molecular Probes) were used to detect the endosomes-EEA1 or lysosomes-LAMP-1 and goat anti-Rat coupled to Alexa-568 was used to detect Giantin-Golgi. Goat anti-mouse Alexa-647 labeled was used to visualize the H-2K^b^/OVA_257–264_ complexes. Dendritic cell surface-derived MHC-I were detected by locating intracellular fluorescent-green punctate dots present. Fluorescence was visualized by ICM using the 488-nm (green) 568-nm (red) and 633-nm (blue) laser lines for excitation of the appropriate fluorochromes. Data were analyzed using ImageJ.1 to select single slices and Adobe Photoshop 9.0 to merge images obtained upon excitation of fluorochromes obtained by red, green and blue channels. Co-localization of three different molecules was evaluated by the presence, intensity and distribution of the white color resulting from the overlapping of green, red and blue.

For quantification of colocalization, a total of approximately 50 DCs were examined at 60× magnification. Quantitative confocal image analysis was done by single cell identification using Open*lab* software and the relative fluorescent intensity of green, red, blue, yellow, purple, light blue and white pixels was assessed. The relative fluorescent intensity of all individual colors was then expressed as percent of total fluorescence intensity.

### *In vitro* cross-presentation and T cell proliferation

Primary DCs were isolated from bone marrow precursors of K^b^WT, Δ7, and ΔY transgenic mice, by culturing *in vitro* in X63-Ag8-plasmacytoma-derived GM-CSF (gift from David Gray, University of Edinburgh, UK) at 20 µL/10 mL of RPMI complete media. On day 8, bone marrow derived DCs (bmDCs) were stained with antibodies against H-2K^b^ (AF6.88), I-A^b^ (AF6.120.1) and CD11c (HL3) (Pharmingen) to test their purity then incubated for 6 hours with 10 mg/mL OVA or with bovine serum albumin or synthetic immunodominant peptide OVA_257–264_ as controls. Next, they were washed in cold PBS and fixed in 0.0005% glutaraldehide to preserve surface K^b^/OVA_257–264_ complexes. B3Z T cell hybridoma (kind gift from Nilabh Shastri at UC Berkeley) were labeled with 10 µM CFSE (Molecular Probes) at 37°C for 30 min and 10^5^ cells were co-cultured with OVA-pulsed transgenic mice DCs in 48-well plates at 50∶50 ratio for 24 and 48 hrs. Next, the mixed cells were washed and stained with anti-CD3-PE labeled antibody (BD Pharmingen) to detect the T cells and analyzed by flow cytometry. Data were acquired using FACSCalibur™ and the CFSE fluorescence (FL1) and CD3 (FL2) positive populations of B3Z-T cells were analyzed with FlowJo software to assess their proliferation.

### Statistical Analysis

Student's T test was used to compare the numbers of fluorescently labeled H-2K^b^ in the cytoplasm of transgenic mouse DCs assessed by ICM and the percent down regulation of H-2K^b^ molecules in transgenic mice DCs at 0°C compared to upon incubation at 37°C as measured by FACS. The difference between two populations was considered statistically significant if P<0.05 (two-tailed, two sample equal variance).

## Results

### Constitutive internalization of MHC-I in dendritic cells is differentially affected by cytoplasmic tail mutations

**Figure 1:**
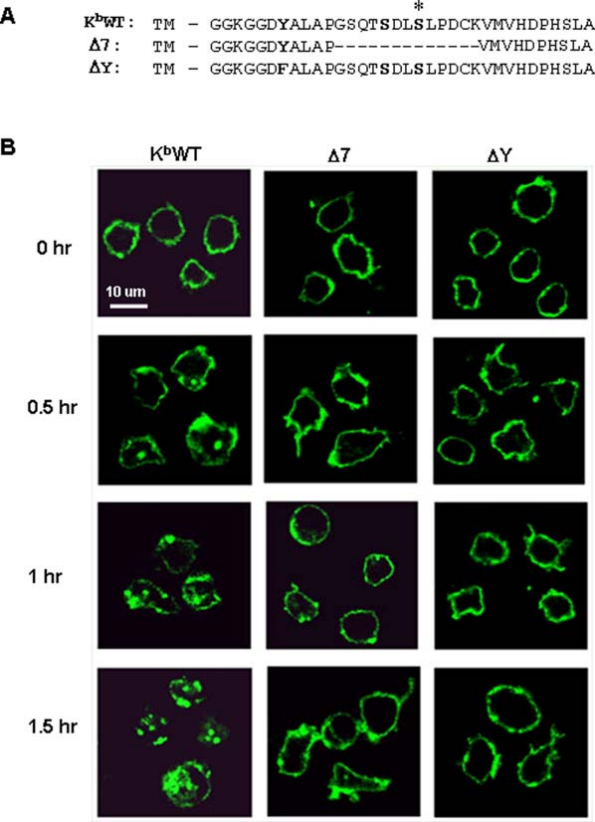
Constitutive MHC-I internalization in DCs is differentially controlled by cytoplasmic tyrosine- and exon 7-dependent mechanisms. (A) Amino acid sequences of the cytoplasmic domains of wild-type H-2K^b^ and the two cytoplasmic tail mutants Δ7 and ΔY. The asterisk denotes a known conserved serine phosphorylation site. The Δ7 mutant contains a deletion of the 13 amino acids comprising exon 7, indicated as dashed lines. Highlighted amino acids indicate conserved tyrosine and serine residues. TM, transmembrane domain. (B) Splenic dendritic cells isolated from K^b^WT, and Δ7 and ΔY transgenic mice were labeled with FITC-conjugated H-2K^b^-specific mAb, washed, and incubated at 37°C for the indicated time points. DCs were then imaged using confocal fluorescence microscopy to visualize internalized MHC-I-containing vesicles. Data are representative of at least 3 images captured from 2 independent experiments.

To elucidate the role of distinct cytoplasmic domain motifs in MHC-I molecular trafficking and function *in vivo*, we generated transgenic mice, described previously [21], expressing either wild type H-2K^b^ (K^b^WT), K^b^ containing a point mutation of the highly conserved exon 6-encoded tyrosine (ΔY), or K^b^ containing a deletion of 13 amino acids encoded by exon 7, including at least one highly conserved serine phosphorylation site (Δ7, Figure 1A). Several distinct founder lines from each of the three strains were obtained by backcrossing the transgenic mice onto a C3H background (haplotype H-2^k^).

To determine whether DCs constitutively internalize surface MHC-I, as has been reported for activated T lymphocytes (22), we performed MHC-I endocytosis experiments on spleen-derived DCs. K^b^-specific, FITC-labeled antibodies were used to stain DCs and internalization of labeled K^b^ molecules was evaluated over time. Fluorescently-labeled intracellular vesicles appeared after 30 min in the K^b^WT transgenic mice DCs and were observed in the majority of DCs examined (Figure 1B). In contrast, DCs from both Δ7 and ΔY transgenic mice showed an almost complete absence of these fluorescent vesicles up to 90 minutes of chase, indicating that spontaneous internalization of K^b^ molecules was abrogated.

**Figure 2:**
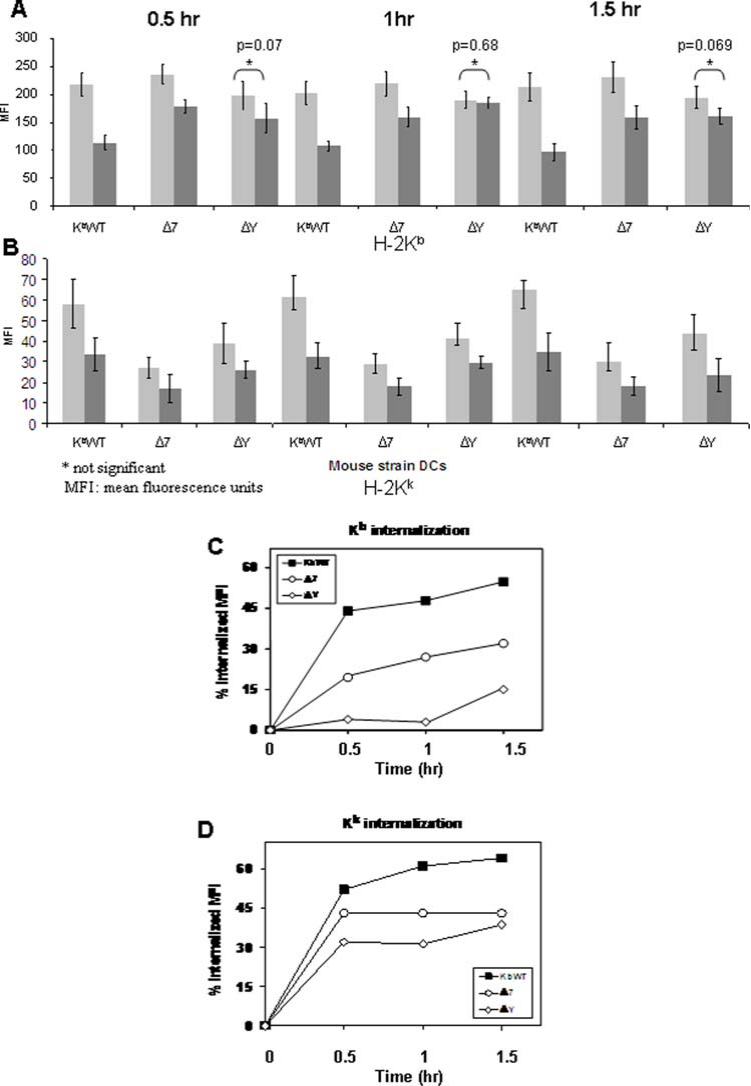
Quantification of MHC-I internalization in DCs. The dynamics of MHC Class I internalization is assessed by the reduced mean fluorescence units of FITC-labeled H-2K^b^ surface expression. Following labeling with (A and C) FITC-conjugated H-2K^b^- or (B and D) PE-conjugated H-2K^k^-specific antibodies and internalization at 37°C for the indicated time points, flow cytometric analysis was conducted to assess internalization of K^b^ and K^k^ molecules, as measured by the reduction in FITC and PE mean fluorescence intensities over time, respectively. Data are representative of 3 different experiments performed in triplicate.

In order to more quantitatively measure MHC-I internalization in DCs, we used flow cytometric analysis following staining with labeled antibodies specific for K^b^ and K^k^. As shown in Figure 2A, 2B, and 2C both K^b^ and K^k^ were internalized very rapidly in DCs derived from K^b^WT mice, with both molecules demonstrating close to 50% internalization at 30 minutes of chase. By contrast, only 4% of labeled K^b^ molecules were internalized from the surface of ΔY-derived DCs over the same time period (Figure 2A and B). As an internal control for MHC-I internalization, these ΔY-derived DCs efficiently internalized endogenously-expressed wild-type K^k^ molecules (33% internalization at 30 minutes of chase), albeit not as rapidly as observed for K^b^WT DCs (Figure 2C). DCs from Δ7 mice showed an intermediate phenotype, with ∼20% of surface K^b^ molecules being internalized at 30 minutes of chase time (Figure 2A and B). These results demonstrate that, while both the Δ7 and ΔY cytoplasmic tail alterations led to impaired MHC-I endocytosis in DCs, point mutation of the single conserved tyrosine residue resulted in a much more pronounced internalization defect compared to complete deletion of the 13 amino acids comprising exon 7.

### Cytoplasmic tail motifs differentially regulate trafficking of internalized MHC-I molecules through endocytic compartments of DCs

**Figure 3:**
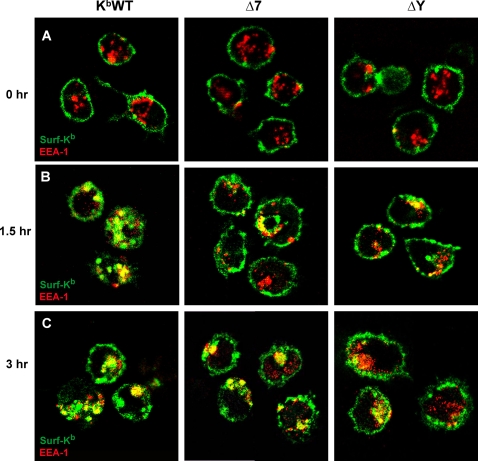
MHC-I cell surface-to-endosome trafficking in DCs is differentially abrogated by mutations in cytoplasmic tyrosine or exon 7-encoded determinants. (A to C) Splenic DCs isolated from K^b^WT, and Δ7 and ΔY transgenic mice were mounted on coverslips, labeled with FITC-conjugated H-2K^b^-specific mAb, washed, then incubated at 37°C for the indicated times. DCs were then fixed, permeabilized and counterstained for EEA-1. Images were acquired using a multiphoton fluorescence confocal microscope. Yellow color indicates co-localization of surface-derived H-2K^b^ (green) with EEA-1 (red). Data are representative of at least 3 images captured from 2 independent experiments.

**Figure 4:**
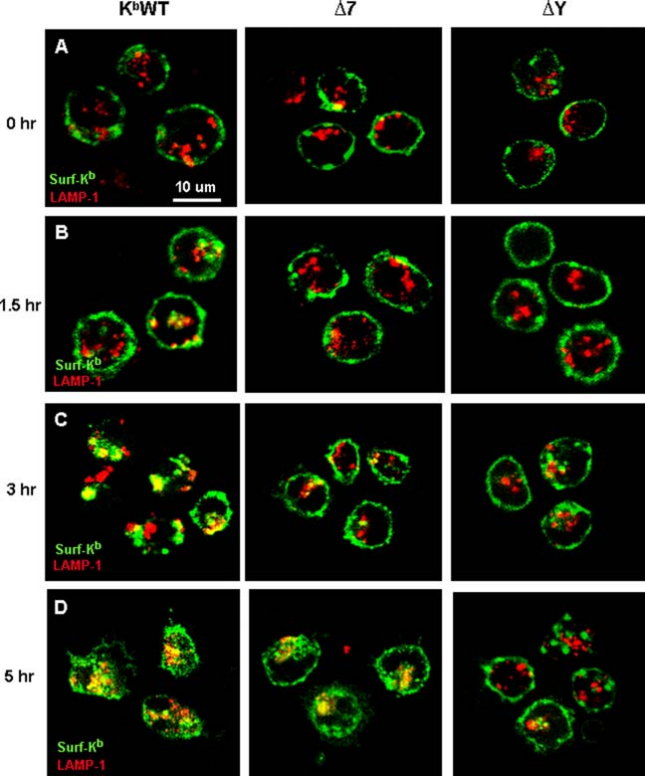
MHC-I cell surface-to-lysosome trafficking in DCs is impaired by mutation in the cytoplasmic tyrosine residue. (A to D) Splenic DCs isolated from K^b^WT, and Δ7 and ΔY transgenic mice were mounted on coverslips, labeled with FITC-conjugated H-2K^b^-specific mAb, washed, and incubated at 37°C for the indicated times. DCs were then fixed, permeabilized, and counterstained for LAMP-1. Yellow color indicates co-localization of surface-derived H-2K^b^ (green) with LAMP-1 (red). Data are representative of at least 3 images captured from 2 independent experiments.

In order to investigate which intracellular compartments K^b^ molecules traffic through following internalization from the DC surface, spleen-derived DCs from all transgenic mice were initially surface-labeled at 4°C with K^b^-specific mAbs. Following washing and incubation at 37°C to induce MHC-I internalization, DCs were fixed and permeabilized at different time points and stained with antibodies specific for EEA-1, a marker of early endosomes, or LAMP-1, a marker of late endosomes and lysosomes. Both wild-type and exon 7-mutated K^b^ molecules showed significant colocalization with early endosomal markers at 1.5 and 3 hours of chase. By contrast, ΔY molecules showed minimal or no overlap with early endosomes at those time points (Figures 3A to 3C). Notably, surface ΔY molecules were also largely excluded from LAMP-1-positive late endosomes and lysosomes, even at 5 hours of chase (Figures 4A to 4D). By contrast, co-localization of surface-derived wild-type K^b^ molecules with LAMP-1 became evident after only 90 minutes of incubation at 37°C (Figure 4B). Interestingly, although little or no colocalization of Δ7 molecules with LAMP-1 could be observed at earlier time points, significant overlap could be detected at five hours of chase (Figure 4B to 4D).

Taken together, these data indicate that while K^b^ molecules lacking exon 7 show significantly delayed internalization kinetics compared with wild-type K^b^ molecules, they nonetheless can traffic from the cell surface into both early and late endosomal/lysosomal compartments of DCs. In contrast, tyrosine point-mutated K^b^ molecules were almost completely abrogated in their ability to traffic from the cell surface to endosomal/lysosomal compartments containing either EEA1 or LAMP-1.

### Cytoplasmic tail mutations reveal distinct pathways of MHC-I trafficking through exogenous peptide-loading compartments of DCs

**Figure 5:**
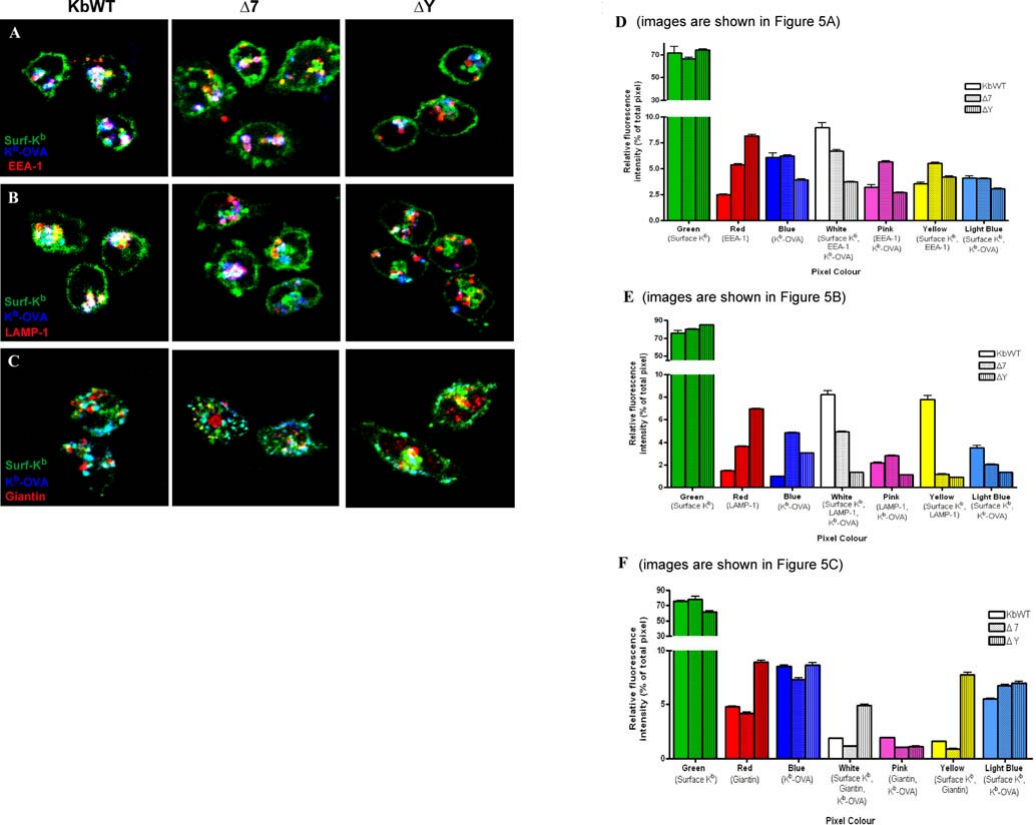
Cytoplasmic tail mutations significantly reduce the contribution of surface MHC-I molecules to endosomal and lysosomal peptide-loading compartments. (A to C) Splenic DCs isolated from K^b^WT, Δ7, and ΔY transgenic mice were labeled with FITC-conjugated H-2K^b^-specific mAb, washed and incubated for 6 hr at 37°C in 5 mg/mL ovalbumin protein. DCs were then labeled with mAbs specific for (A) early endosomal antigen, EEA-1, (B) lysosomal marker LAMP-1 and (C) Golgi marker Giantin. All DCs were simultaneously co-stained with purified 25.D1.16 (anti-H-2K^b^/OVA_257–264_) antibody. Cellular markers EEA-1, LAMP-1 and Giantin were visualized by staining with secondary antibodies coupled to Alexa-568 (red) whereas H-2K^b^/OVA_257–264_ complexes were visualized by staining with secondary antibody conjugated to Alexa-647 (blue). Three-color fluorescence was detected by laser scanning confocal microscopy of 488-nm (green), 568-nm (red), and 633-nm (blue) wavelengths. Photographs depict three-color image overlays to assess colocalization of the three markers. White color indicates a triple overlap of all three markers (green+red+blue), whereas yellow (green+red), pink (red+blue), and light blue (green+blue) indicate overlap of two of the three markers. D to F shows a quantitative assessment of internalized MHC-I and MHC-I/peptide complexes within intracellular compartments of DCs. Three-color confocal overlay images of DCs, as shown in Figure 5, were analyzed for relative fluorescent color (pixel) intensity in order to obtain a quantitative measure of fluorophore colocalization. For each data set, 30 to 50 individual DCs derived from each of the indicated mouse strains were analyzed. The green color indicates surface-labeled H-2K^b^, the blue color indicates K^b^-OVA_257–264_ peptide complexes, and red color indicates either (A) early endosomal antigen (EEA-1), (B) LAMP-1, or (C) Giantin. White pixels indicate triple overlap of all three markers (green+red+blue), whereas yellow (green+red), pink (red+blue), and light blue (green+blue) indicate overlap of two of the three markers. Graph depicts individual color pixel mean percentages and standard deviations, as calculated by dividing the number of pixels of a given color by the total number of colored pixels counted. Data are representative of at least 3 images captured from 4 independent experiments.

Prior to assessing the entry of surface-derived K^b^ molecules into endocytic compartments, a competition assay using flow cytometry was designed. No change in H-2K^b^ binding was observed with sequential staining of K^b^ molecules preceded or followed by staining of H-2K^b^/OVAp complexes (refer to Supporting Information: Text S1 and Figure S1). The same was observed for the H-2K^b^/OVAp surface expression indicating that there was no competition of these antibodies for binding to specific sites. In order to assess how entry of surface-derived K^b^ molecules into endocytic compartments coincides with exogenous antigen loading, K^b^ surface-labeled DCs were pulsed with soluble OVA protein for 6 hours to allow simultaneous K^b^-FITC internalization and OVA uptake and antigen processing. DCs were then fixed, permeabilized and stained with fluorescently-labeled antibodies specific for either EEA1, LAMP-1, or Giantin, a Golgi marker (red) in addition to 25.D1, an antibody that specifically stains K^b^/OVA_257–264_ complexes (blue). Stained DCs were then examined and imaged by confocal microscopy to assess colocalization of the three fluorophores. In order to obtain a more precise quantification of this colocalization, we used image analysis software to obtain pixel counts for the seven colors present in each overlaid confocal image [green, red, blue, yellow (g+r), light blue (g+b), pink (r+b), and white (g+r+b)]. While Figure 5A, B and C shows confocal DC images representative of each mouse strain, quantification of fluorescence intensity was performed on images from a further 30 to 50 individual DCs from each mouse strain in order to generate the quantitative data summarized in Figure 5 (D to F).

When DCs from all transgenic mice strains were incubated at 4°C, no loading of OVA peptide antigen (blue) was detectable and K^b^ molecules remained at the cell surface, as expected (not shown). Upon incubation at 37°C for 6 hrs, both surface K^b^ and K^b^-OVA complexes showed abundant colocalization (white) with EEA1-positive intracellular compartments in K^b^WT-derived DCs (Figure 5A). Notably, overlapping of EEA-1 and K^b^-OVA complexes without surface K^b^ (indicated by pink color) was significantly higher (p = 0.002) in Δ7-derived DCs (Figure 5D). By contrast, early endosomes from ΔY-derived DCs contained no detectable surface-derived K^b^ or K^b^-OVA complexes (Figure 5A).

Similarly, K^b^WT-derived DCs demonstrated extensive triple overlap of surface-derived K^b^ and OVA-loaded K^b^ molecules with LAMP-1-positive compartments (Figure 5A). By comparison, DCs derived from Δ7 and ΔY mice showed 2-fold (p = 0.004) and 6-fold (p = 0.0001) less triple colocalization, respectively (Figures 5A and 5E). Furthermore, in K^b^WT-derived DCs the majority (>80%) of LAMP-1-positive compartments also contained surface-derived K^b^, with ΔY-derived DCs showing approximately 5-fold less (p = 0.0001) overlap of these two markers and Δ7-derived DCs demonstrating an intermediate phenotype (Figure 5E). Conversely, ΔY-derived DCs showed a strikingly higher (p = 0.006) percentage of LAMP-1 alone (red pixels, not colocalized with the other two markers) compared with K^b^WT-derived DCs.

Co-staining with the Golgi marker Giantin showed very little colocalization of surface K^b^ or K^b^-OVA complexes with Golgi in DCs from K^b^WT and Δ7 mice. Interestingly, in ΔY-derived DCs, Giantin and surface K^b^ demonstrated a significant (p = 0.0004) degree of overlap (yellow) and some triple colocalization (white) (p = 0.007) was also apparent (Figures 5C and 5F).

In summary, while surface K^b^WT molecules appear to form an abundant source of MHC-I molecules for early and late endosomal peptide-loading, surface ΔY molecules do not gain access to these peptide-loading sites due to aberrant intracellular trafficking. Surface K^b^ molecules that lack exon 7, by contrast, appear to traffic readily into early endosomal compartments, but are significantly delayed in their ability to traffic into late endosomes or lysosomes. Taken together, these data reveal distinct intracellular trafficking patterns for K^b^WT, Δ7 and ΔY molecules in DCs that may provide a plausible mechanism to explain their different abilities to cross-present exogenous antigens.

### Cross-presentation and T cell activation is impaired in ΔY-derived DCs following 6 hour incubation with OVA antigen

**Figure 6:**
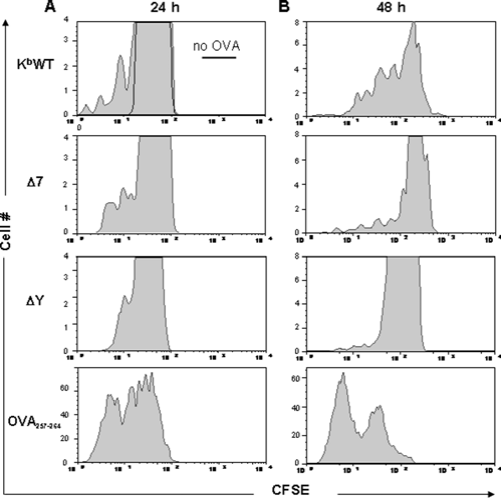
T cell activation is severely compromised in protein-pulsed DCs of transgenic mice containing the cytoplasmic tail mutation. bmDCs from K^b^WT, Δ7 and ΔY transgenic mice were isolated, incubated for 6 hours with 10 mg/mL OVA and co-cultured for 24 and 48 hours with B3Z-T hybridoma cells previously labeled with 1 µM of CFSE. Mixed cultures were then stained with anti-CD3-PE antibody and flow cytometry was conducted to examine the CFSE/CD3^+^ T cell population and proliferation. Histograms depict proliferating T cells following incubation with OVA-pulsed DCs (including one representative of OVA_257–264_) of transgenic mice for 24 (A) and 48 (B) hours. Data are representative of 1 experiment performed in triplicate.

To correlate the impaired intracellular trafficking of MHC Class I molecules in our transgenic mouse DCs with functional impairment of OVA cross-presentation and T cell activation during the 6 hour time period, T cell proliferation was monitored for 48 hours following a 6 hour incubation of transgenic mouse DCs with OVA protein. Proliferation of CFSE-labeled T cells was detectable following 24 hours of co-incubation with K^b^WT transgenic OVA-pulsed DCs (Figure 6A). However, fewer T cells proliferated following incubation with Δ7-derived DCs while this was barely measurable following co-culture with ΔY-DCs. After 48 hours, more then half of the T cells incubated with K^b^WT-DCs had proliferated and although less proliferation was detected still a considerable fraction of T cells incubated with Δ7-DCs were dividing. However, when T cells were incubated with ΔY-DCs, over 90% of them did not proliferate indicating that cross-presentation of OVA antigen and T cell activation was severely impaired presumably due to defective internalization and intracellular trafficking of surface ΔY-K^b^s. No difference in T cell activation was observed, between transgenic mice DCs following their incubation with OVA_257–264_.

## Discussion

Although cross-presentation by DCs is a crucial prerequisite for effective priming of cytotoxic T-cell responses against bacterial, viral, and tumor antigens *in vivo*, several mechanistic aspects of these pathways remain poorly defined. The results presented here delineate constitutive pathways of MHC-I intracellular transport in DCs, and illuminate the nature of the intracellular compartments of DCs where peptides from exogenously-derived antigens are loaded for cross-presentation. Importantly, the internalization data demonstrate that MHC Class I from the cell surface can enter a vesicular compartment that contains also complexes of MHC Class I and cross-presented antigen.

Our microscopic analysis of spleen-derived DCs reveals for the first time, that wild-type (WT) surface MHC-I molecules are internalized and transported to early and late endosomal/lysosomal compartments, where peptides from exogenously-derived antigens can be loaded [13], [18], [21], [22]. While efficient MHC Class I loading with exogenous antigen has been shown to occur in early endosomes (13), some endosome-to-lysosome trafficking or exchange of MHC Class I containing antigenic peptide, can not be excluded particularly at later time points (6 hr) that could be occurring during fusion of endocytic compartments while processing the exogenous antigen. However, in this model, it is difficult to assess the relevance of MHC Class I that contain antigenic peptide in late endosomes for priming T cells. Our functional data indicate that peptide-loaded K^b^ molecules, can reach the cell surface within 6 hours, and are sufficient to induce T cell proliferation. While microscopy could somewhat visualize the peptide-loaded K^b^s on the cell surface, this was not convincingly quantifiable and therefore the assessment of T cell activation complemented the previous observations and revealed the relevance of their presence on the cell surface within the time frame assessed. Also, our model including the 6 hour time frame, tends to validate the vacuolar pathway of antigen presentation since it allows little time for the antigen to access the cytosol, although as shown by microscopy and some minimal T cell activation in ΔY-DCs, this could occur even at early time points. Consequently, it is reasonable to assume that this pathway may account for the first wave of T cells that are generated following antigen exposure during the immune response. Importantly in the present study we can clearly demonstrate that this pathway is dependent on the conserved cytoplasmic tyrosine residue encoded by exon 6, confirming its role as part of a tyrosine-based endocytic-sorting motif (YXXA) that controls endocytic trafficking of surface MHC-I molecules [23]. It is thus likely that the previously reported cross-presentation deficiency in ΔY-derived DCs resulted from the inability of these tyrosine-mutated surface K^b^ molecules to traffic through endocytic loading compartments (ELCs) [21].

Our data also demonstrates a distinct role for exon 7-encoded amino acids in controlling MHC-I intracellular trafficking in DCs. The existence of exon 7-deleted MHC-I isoforms that lack conserved serine phosphorylation sites has been reported in several species including mouse, arising in different cell types to varying degrees as a result of differential RNA splicing [24]. As has previously been reported for lymphoblastoid cell lines, exon 7-deleted (Δ7) MHC-I molecules in DCs show significantly delayed kinetics of internalization from the cell surface [25]. However, unlike ΔY, surface Δ7 molecules do possess the ability to traffic though both early and late endosomal compartments, albeit more slowly than K^b^WT molecules. Delayed cell surface internalization may actually provide an advantage by prolonging antigen presentation to augment CTL priming, a notion consistent with our previous observation that Δ7 mice consistently generated more vigorous antiviral CTL responses compared to K^b^WT mice [21].

The distinct intracellular localizations of newly-formed K^b^-OVA complexes observed in K^b^WT, Δ7, and ΔY-derived DCs support the emerging concept of multiple pathways of peptide loading of cross-presented antigens [26], [27]. In our study, the majority of K^b^WT-OVA complexes were found within early and late endosomal/lysosomal compartments of DCs after exposure to OVA. Like K^b^WT, the majority of Δ7-OVAp complexes were also found within early and late endosomes. By contrast, ΔY-OVAp complexes were comparatively less abundant, and those observed were almost exclusively in non-endolysosomal compartments, with some appearing in the Golgi and others colocalizing with the ER marker Grp78 (unpublished).

Our data suggests that the vacuolar pathway of exogenous antigen loading plays a principal role in cross-presentation, having observed the majority of K^b^WT-OVA complexes within early and late endosomes/lysosomes of DCs following addition of soluble ovalbumin. However, the existence of K^b^-OVA complexes within non-endolysosomal compartments, as seen in both Δ7 and ΔY DCs, suggests that a second compartment of exogenous antigen loading also exists. These observations could support the endosome-to-cytosol pathway, in which exogenous antigens are extruded into the cytosolic pool of protein antigens and are processed by proteosomes before being transported into the ER for loading onto nascent MHC-I molecules. However, co-localization analysis of K^b^-OVA complexes with the ER marker Grp78 showed that only a minor fraction of Grp78-positive compartments contained such complexes, and these compartments also colocalized with surface-derived K^b^ molecules (unpublished data). This could be interpreted as evidence of “ergosomal” peptide loading, in which the relevant loading sites consist of mixed compartments containing markers of both ER and endolysosomes [28]. While it remains disputed whether phagosomal membranes are ER- or plasma membrane-derived [16], it is possible that cross presentation-competent, ergosomal organelles may be formed through another mechanism of vesicular fusion, following endocytosis. Further studies utilizing different ER specific markers will need to be performed before definitive conclusions can be reached.

**Figure 7:**
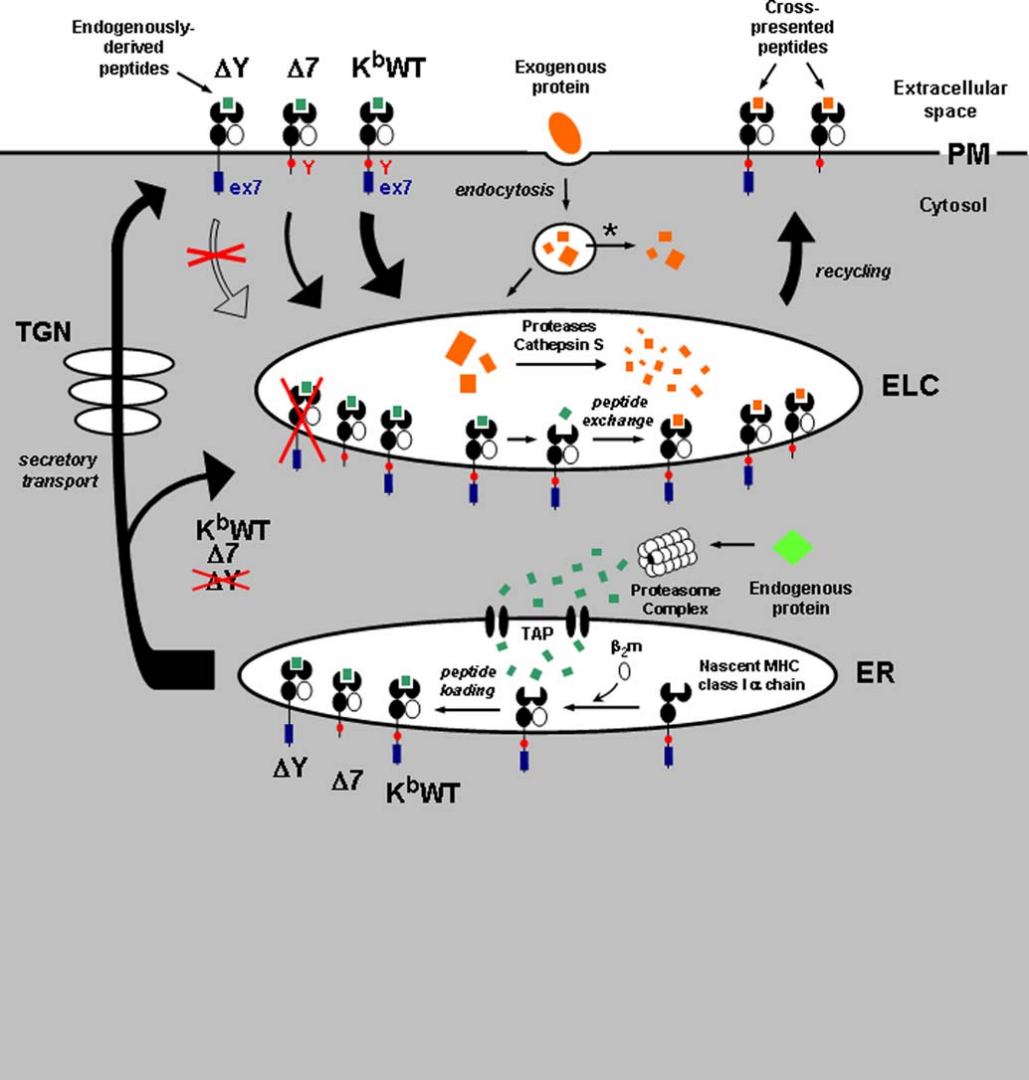
Model of dendritic cell MHC-I trafficking and cross-presentation. Schematic representation of trafficking routes for K^b^WT, Δ7, and ΔY molecules in dendritic cells, depicting proposed intracellular sites of antigen acquisition. In the direct presentation pathway *(bottom)*, endogenously-synthesized proteins *(green)* are degraded by cytosolic proteosome complexes into antigenic peptides, which are transported by the transporter associated with antigen processing (TAP) into the endoplasmic reticulum (ER) for binding to nascent class Iα chain/β2-microglobulin dimers. K^b^WT, Δ7, and ΔY molecules initially loaded in the ER with endogenously-derived peptides *(green)* are then transported through the cis- and trans-Golgi (TGN) and to the cell surface via the secretory pathway. Alternatively, a subset of K^b^WT and Δ7, but not ΔY, molecules may be re-routed from the secretory pathway directly into endolysosomal compartments, although this pathway remains largely uncharacterized. In cross-presentation, exogenous protein antigens *(orange, top)* are internalized into endocytic vesicles, where they can be transported into the cytosol via ER-associated retrotranslocation (asterisk) and subsequently enter the direct presentation pathway. Exogenous antigens may also be transported into endolysosomes, where they are degraded by resident proteases and Cathepsin S into antigenic peptides *(orange)*. Surface MHC-I molecules are constitutively internalized and transported through the endocytic pathway by a mechanism that requires the MHC-I cytoplasmic tyrosine (Y) residue. MHC-I molecules lacking Exon 7 (Ex7), despite abundant colocalization within endosomes and lysosomes, are significantly delayed in their endocytic transport. MHC-I transport through early endosomes and late endosomes/lysosomes seems to be required for acquisition and cross-presentation of exogenously-derived peptides, suggesting that such peptides are bound to recycling MHC-I molecules directly within endocytic loading compartments (ELCs).

Collectively, our results support the model of DC MHC-I trafficking and exogenously-derived peptide loading depicted in Figure 7. In this model, K^b^WT, Δ7, and ΔY molecules initially appear in the ER as nascent MHC-I heavy chains that bind to β_2_-microglobulin and are loaded primarily with endogenously-derived peptides generated by the proteosome and transported by TAP. After secretory transport through the Golgi and to the cell surface, wild-type K^b^ molecules are rapidly and efficiently internalized and transported to ELCs, where peptide exchange and loading of peptides from exogenously-derived antigens generated by endolysosomal proteases and cathepsins may occur. These newly-loaded MHC-I complexes are then transported by an undefined mechanism back to the cell surface for cross-presentation to T cells. By contrast, K^b^ molecules lacking exon 7 are internalized and transported to ELCs, but significantly more slowly than wild-type molecules. Interestingly, this delayed kinetics does not seem to significantly reduce the level of Δ7 molecules residing within ELCs. This raises the possibility that MHC-I molecules might gain access to endolysosomal compartments by means other than internalization from the cell surface. The relative abundance of Δ7-OVA complexes in ELCs that are not colocalized with surface-derived Δ7 molecules (Figures 5A and 5B, pink color) supports this notion. This may indicate loading of newly-synthesized MHC-I molecules through the classical pathway and subsequent transport to endosomes, or alternatively may represent loading of nascent K^b^ molecules within ELCs. MHC-I molecules may conceivably traffic to ELCs directly from the ER to create ergosomal mix/fusion compartments, but this pathway is not currently well understood [29]. Another potential trafficking route may involve sorting of MHC-I molecules from the trans-Golgi into endolysosomes, a pathway that has been well-described for other transmembrane proteins [30]. It is tempting to speculate that the ΔY molecules we observed in Golgi compartments of DCs may be trapped there as a result of their inability to interact with the appropriate tyrosine motif-binding adaptin proteins to be sorted into clathrin-coated vesicles destined for ELCs [31].

While the results presented here provide an important first step for delineating intracellular routes of MHC-I trafficking in DCs and defining the molecular mechanisms that contribute to cross-presentation, many questions remain unresolved. For example, it will be interesting to examine whether serine phosphorylation is the major mechanism that modulates MHC-I internalization and recycling, and whether tyrosine phosphorylation also plays a role [32]. DC maturation stimuli (ie. TLR ligands) that induce changes in MHC-I molecular trafficking and remodeling of intracellular compartments will also shed light on how specialized exogenous antigen loading can occur with maximum efficiency [33]. Finally, there are unresolved questions regarding the importance of antigen entry route on cross-presentation. Soluble ovalbumin has recently been shown to be dependent upon the mannose receptor for its uptake into DCs (13). It will be interesting to determine whether the MHC-I cytoplasmic domain plays a similar role in the cross-presentation of pinocytosed or phagocytosed antigens as well as the MHC Class I targeting in the organelle where loading takes place following distinct routes of uptake'. With growing evidence that cross-presentation is crucial for the generation of appropriate CD8^+^ immune responses, future work should focus on uncovering the relevant mechanisms and better characterizing the dynamic nature of cross-presentation compartments. Understanding these issues will ultimately lead to the development of more potent dendritic cell-based vaccines for cancer and other diseases, as well as novel strategies to target endogenous DCs *in vivo* to generate optimal CD8^+^ T-cell priming against defined antigens.

## Supporting Information

Text S1 (0.02 MB DOC)

Figure S1 DC2.4 dendritic cells were incubated with 1 µM OVA_257–264_ or PBS and labeled sequentially with anti-H-2K^b^-FITC followed by anti H-2K^b^/OVA_257–264_ antibodies and vice versa. Flow cytometry was conducted to assess the H-2K^b^ and H-2K^b^/OVA_257–264_ complexes. Data represents one experiment.
(0.06 MB TIF)
