# Supplementary material for: MHC Class I Endosomal and Lysosomal Trafficking Coincides with Exogenous Antigen Loading in Dendritic Cells
Source: PLoS One. 2008 Sep 19;3(9):e3247. doi: 10.1371/journal.pone.0003247 (PMC2532750; doi:10.1371/journal.pone.0003247)
Supplement: Text S1 — (0.02 MB DOC) [file pone.0003247.s001.doc]

**SUPPORTING INFORMATION - Text S1**

**Materials and Methods**

Prior to the examination of the intracellular trafficking of surface MHC Class I, a competition experiment was performed to exclude H-2K^b^ antibody binding in competition for H-2K^b^/OVAp binding. For this, DC2.4 dendritic cells (gift from Kenneth L Rock from University of Massachusetts) were incubated with 1 µM OVA_257-264_ or PBS for 1 hr at 37ºC. After washing, DCs were labeled sequentially with anti 25.D1.16 (anti-H-2K^b^/OVA_257-264_) antibody followed by H-2K^b^-FITC and *vice versa* including the isotype control IgG1-PE (BD Biosciences, Mississauga, ON, Canada ) of H-2K^b^/OVAp antibody for 30 min at 4°C. FACSCalibur™ was used to assess H-2K^b^ and H-2K^b^/OVAp binding following sequential staining to evaluate differences that would indicate competition for antibody binding sites.

**Results**

Prior to assessing the entry of surface-derived K^b^ molecules into endocytic compartments, a competition assay using flow cytometry was designed. No change in H-2K^b^ binding was observed with sequential staining of K^b^ molecules preceded or followed by staining of H-2K^b^/OVAp complexes (Figure S1). The same was observed for the H-2K^b^/OVAp surface expression indicating that there was no competition of these antibodies for binding to specific sites.

**Figure Legend**

**Figure S1.**

DC2.4 dendritic cells were incubated with 1 µM OVA_257-264_ or PBS and labeled sequentially with anti-H-2K^b^-FITC followed by anti H-2K^b^/OVA_257-264_ antibodies and *vice versa*. Flow cytometry was conducted to assess the H-2K^b^ and H-2K^b^/OVA_257-264_ complexes. Data represents 1 experiment.
