# Supplementary figures and images for: MHC Class I Endosomal and Lysosomal Trafficking Coincides with Exogenous Antigen Loading in Dendritic Cells
Source: PLoS One. 2008 Sep 19;3(9):e3247. doi: 10.1371/journal.pone.0003247 (PMC2532750; doi:10.1371/journal.pone.0003247)

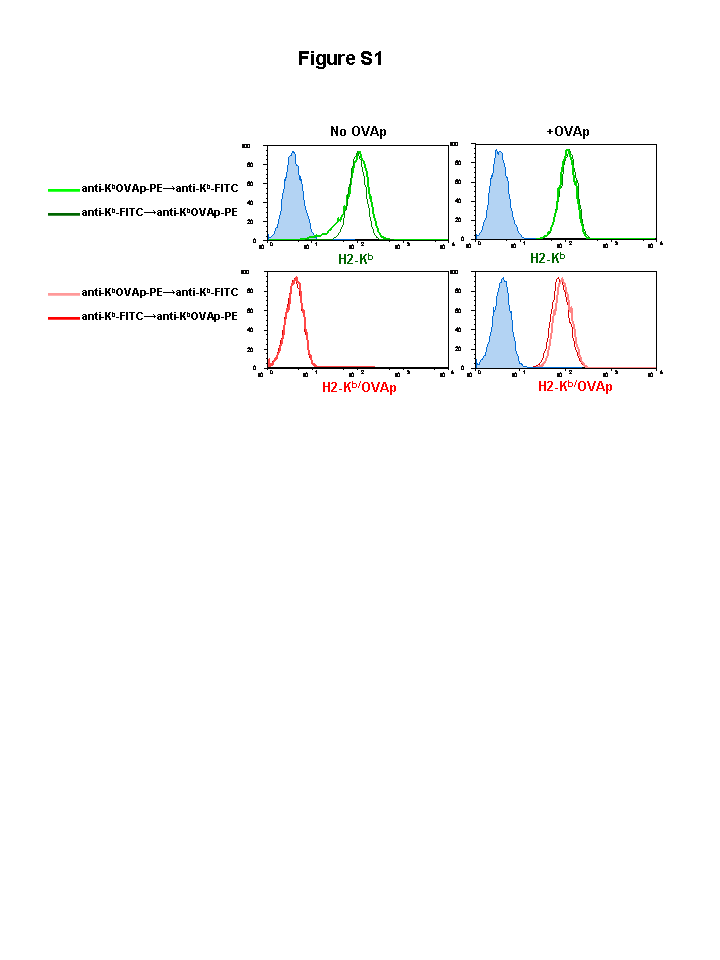

Supplement: Figure S1 — DC2.4 dendritic cells were incubated with 1 µM OVA257–264 or PBS and labeled sequentially with anti-H-2Kb-FITC followed by anti H-2Kb/OVA257–264 antibodies and vice versa. Flow cytometry was conducted to assess the H-2Kb and H-2Kb/OVA257–264 complexes. Data represents one experiment. (0.06 MB TIF) [file pone.0003247.s002.tif]
